# Shear-Stress-Gradient and Oxygen-Gradient Ektacytometry in Sickle Cell Patients at Steady State and during Vaso-Occlusive Crises

**DOI:** 10.3390/cells11030585

**Published:** 2022-02-08

**Authors:** Camille Boisson, Elie Nader, Céline Renoux, Alexandra Gauthier, Solène Poutrel, Yves Bertrand, Emeric Stauffer, Emilie Virot, Arnaud Hot, Romain Fort, Giovanna Cannas, Philippe Joly, Philippe Connes

**Affiliations:** 1 Laboratoire Interuniversitaire de Biologie de la Motricité (LIBM) EA7424, Team “Vascular Biology and Red Blood Cell”, Université Claude Bernard Lyon 1, 69008 Lyon, France; camille.boisson@chu-lyon.fr (C.B.); elie.nader@univ-lyon1.fr (E.N.); celine.renoux@chu-lyon.fr (C.R.); alexandra.gauthier@ihope.fr (A.G.); solene.poutrel@chu-lyon.fr (S.P.); emeric.stauffer@chu-lyon.fr (E.S.); emilie.virot2@chu-lyon.fr (E.V.); philippe.joly@chu-lyon.fr (P.J.); 2 Laboratoire d’Excellence du Globule Rouge (Labex GR-Ex), PRES Sorbonne, 79015 Paris, France; 3 Laboratoire de Biochimie et de Biologie Moléculaire, UF de Biochimie des Pathologies Erythrocytaires, Centre de Biologie et de Pathologie Est, Hospices Civils de Lyon, 69500 Bron, France; 4 Institut d’Hématologique et d’Oncologique Pédiatrique, Hospices Civils de Lyon, 69008 Lyon, France; yves.bertrand@ihope.fr; 5 Service de Médecine Interne, Hôpital Edouard Herriot, Hospices Civils de Lyon, 69008 Lyon, France; arnaud.hot@chu-lyon.fr (A.H.); giovanna.cannas@chu-lyon.fr (G.C.); 6 Service d’Explorations Fonctionnelles Respiratoires-Médecine du Sport et de l’activité Physique, Hospices Civils de Lyon, 69004 Lyon, France; 7 Service de Réanimation Médicale, Hôpital Lyon Sud, Hospices Civils de Lyon, 69495 Pierre-Bénite, France; romain.fort@chu-lyon.fr

**Keywords:** sickle cell disease, red blood cell deformability, oxygen gradient ektacytometry, vaso-occlusive crisis

## Abstract

Oxygen gradient ektacytometry (oxygenscan) measures the changes in red blood cell (RBC) deformability in normoxia and during deoxygenation. We investigated the changes in RBC deformability, measured by both oxygenscan and classical shear-stress-gradient ektacytometry, in 10 patients with sickle cell disease (SCD) during vaso-occlusive crisis (VOC) versus steady state. Oxygenscan and shear-stress-gradient ektacytometry parameters were also measured in 38 SCD patients at steady state on two different occasions. Shear-stress-gradient ektacytometry parameters, maximal RBC deformability at normoxia and the minimum RBC deformability during deoxygenation were lower during VOC compared to steady state. The oxygen partial pressure at which RBCs started to sickle (PoS) was not significantly affected by VOC, but the results were very heterogeneous: the PoS increased in 5 in 10 patients and decreased in 4 in 10 patients. Both oxygenscan and shear-stress-gradient ektacytometry parameters remained unchanged in patients at steady state between two sets of measurements, performed at 17 ± 8 months intervals. In conclusion, the present study showed that both oxygen gradient ektacytometry and shear-stress-gradient ektacytometry are sensitive to disease activity in SCD, and that both techniques give comparable results; however, the oxygen-dependent propensity of RBCs to sickle was highly variable during VOC.

## 1. Introduction

Red blood cell (RBC) deformability is severely decreased in patients with sickle cell disease (SCD, [1,2]). This loss of deformability is the consequence of the presence of an abnormal hemoglobin (hemoglobin S), which may polymerize under deoxygenation (Eaton et al., Blood 1987), causing a mechanical distortion of RBCs (i.e., sickling) [3]. In addition, the repetition of sickling–unsickling episodes causes permanent damages to the RBC membrane [4,5] and is accompanied by cell dehydration [6], which further alter the deformability of the cells. The decreased RBC deformability participates in the occurrence of major complications of SCD, including vaso-occlusive crises (VOC), as well as chronic organ damages [2].

Patients with the largest reduction in RBC deformability at steady state are also those with the most severe anemia and high hemolytic rate, because rigid RBCs are more fragile than deformable RBCs [7]. Consequently, SCD patients who develop complications usually attributed to chronic hemolysis (such as recurrent priapism, cerebral vasculopathy, glomerulopathy or leg ulcers) have greater reduction in RBC deformability than patients without any of these complications [8,9,10,11]. In contrast, SCD patients prone to developing frequent VOCs have greater RBC deformability at steady state (although still reduced compared to healthy controls) than those with few VOCs [12,13]. Better RBC deformability is accompanied by a lower hemolytic rate, less severe anemia and greater blood viscosity, all these factors increasing the risk for VOC [12,14,15]. Indeed, the variability in the reduction in RBC deformability in SCD modulates the clinical heterogeneity of the patients [2]. This variability is related to the presence of highly heterogeneous subpopulations of RBCs with variable degrees of deformability (i.e., reticulocytes, dense RBCs, irreversibly sickled cells …).

Recently, oxygen gradient ektacytometry (oxygenscan) has been proposed as a new possible biomarker of clinical severity in SCD [16,17,18]. In contrast to the classical shear-stress-gradient ektacytometry technique, where RBC deformability is measured in normoxic conditions and at increasing shear stresses (typically from 0.3 to 30 Pa), oxygenscan measures the changes in RBC deformability in normoxic conditions and during deoxygenation while shear stress is kept constant (typically at 30 Pa). It allows the determination of maximal RBC deformability at normoxia (EImax), minimum RBC deformability (EImin) reached at low oxygen partial pressure (pO_2_) and the propensity of RBCs to sickle during deoxygenation (i.e., the pO_2_ at which RBCs start to sickle, called the Point of Sickling (PoS)) [19]. It has been reported that SCD patients with frequent VOC had a higher PoS and lower EImin at steady state than patients with a low VOC rate [17]. In addition, oxygenscan parameters are sensitive to treatment. For instance, hydroxyurea medication decreases PoS and increases EImax and EImin [17]. The same findings have been found recently with voxelotor, a molecule used to increase hemoglobin S affinity to oxygen, to reduce the susceptibility of RBC to sickling during deoxygenation [20]. Indeed, oxygenscan could be a promising new biomarker in SCD [18]. However, no study has investigated the sensitivity of oxygenscan to detect the changes in RBC rheological properties occurring during VOC. In addition, to be considered clinically meaningful, a biomarker needs to be rather stable in patients with no change in their clinical status, clinical management or therapeutics. The aim of the present study was to investigate the changes in RBC deformability measured by oxygen gradient ektacytometry and shear-stress-gradient ektacytometry in SCD patients during VOC compared to steady state, and to test the stability of RBC deformability in SCD patients at steady state.

## 2. Materials and Methods

### 2.1. Patients

Ten SCD patients (8 HbSS and 2 HbSC, 28.1 ± 14.6 years, 3 males/7 females, 7 under hydroxyurea medication) were included in the first part of the study to compare biological markers between clinical steady state and VOC. During VOC, blood was sampled within the first hour of Emergency Department admission before any medication was administered at the hospital, as previously performed in another study [21]. The second part of the study was conducted in 38 SCD patients (29 HbSS, 6 HbSC and 3 HbSβ°, 26.0 ± 14.6 years, 11 males/27 females, 28 under hydroxyurea medication) and compared the biological markers between two periods where they were at steady state (mean time between the two measurements: 17 ± 8 months) with no change in their clinical management and therapeutics. HbSS, HbSC and HbSβ° correspond to patients with homozygous sickle-cell anemia, sickle-hemoglobin C disease and sickle beta 0 thalassemia, respectively. All patients were followed at the Sickle Cell Center of the Academic Hospital of Lyon. Steady state was defined as a period free of any complication and transfusion for at least 3 months. An acute event was considered as a VOC if the painful episode lasted for more than 2 h, the patient felt that the pain was typical of that of vaso-occlusion, no other etiology of pain could be identified by the physicians and the patient was admitted to the Emergency Department to treat the pain [12]. The study was conducted in accordance with the guidelines set by the Declaration of Helsinki and was approved by the Regional Ethics Committees (L14-127).

### 2.2. Hematological and Biochemical Parameters

Hemoglobin concentration (Hb), hematocrit (Hct), mean cell volume (MCV) and mean corpuscular hemoglobin concentration (MCHC) were determined with a hematology analyzer (Advia, Siemens, Rungis, France). C-reactive protein (CRP) and fibrinogen levels were determined by standard biochemical methods.

### 2.3. Shear Gradient Ektacytometry

Ektacytometry was carried out with a laser-assisted optical rotational red-cell analyzer (Lorrca MaxSis, RR Mechatronics, Zwaag, The Netherlands). Twenty-five microliters of EDTA blood were added to 5 mL of an iso-osmolar polyvinylpyrrolidone solution with a mean viscosity of 27–33 cP (Mechatronics, The Netherlands) and osmolality of 284–304 mOsm/kg. The Elongation Index (EI) was calculated from the diffraction pattern collected by the camera of the LORRCA Maxsis and reflected RBC deformability [22]. EI data were obtained at 37 °C in normoxic conditions and at 9 increasing shear stresses (0.3, 0.53, 0.95, 1.69, 3, 5.33, 9.49, 16.87 and 30 Pa), as previously described [12,13,23]. Measurements were performed less than 4 h after blood sampling.

### 2.4. Oxygen Gradient Ektacytometry

Oxygen gradient ektacytometry was carried out with the oxygenscan module of the LORRCA Maxsis to measure RBC deformability over an oxygen gradient. A volume of 50 µL of blood, standardized to a fixed RBC count of 200 × 10^6^, was mixed with 5 mL of Oxy-Iso polyvinylpyrrolidone (PVP) suspension with a mean viscosity of 28–30 cP (Mechatronics, The Netherlands) and osmolality of 282–286 mOsm/kg. The suspension was sheared at 30 Pa and 37 °C while the oxygen partial pressure (pO_2_) was gradually decreased from 160 mmHg to 20 mmHg (deoxygenation) and then returned to normoxic values [16,17,19]. The diffraction pattern was analyzed by the computer to calculate EImax and EImin, and to determine the PoS values. All measurements were standardized as recommended [17,24,25]. Measurements were performed less than 4 h after blood sampling.

### 2.5. Statistics

A paired Student’s *t-*test was used to compare the biological parameters in the same individuals between steady state and VOC, and in the same individuals between the two times of measurements. Pearson correlations were performed to test the associations between different parameters. The significance level was defined as *p* < 0.05. Data are displayed as means ± SD. Statistical analyses were conducted using SPSS software (version 20, IBM SPSS Statistics, Chicago, IL, USA).

## 3. Results

### 3.1. Steady State vs. VOC Comparisons

**Figure 1:**
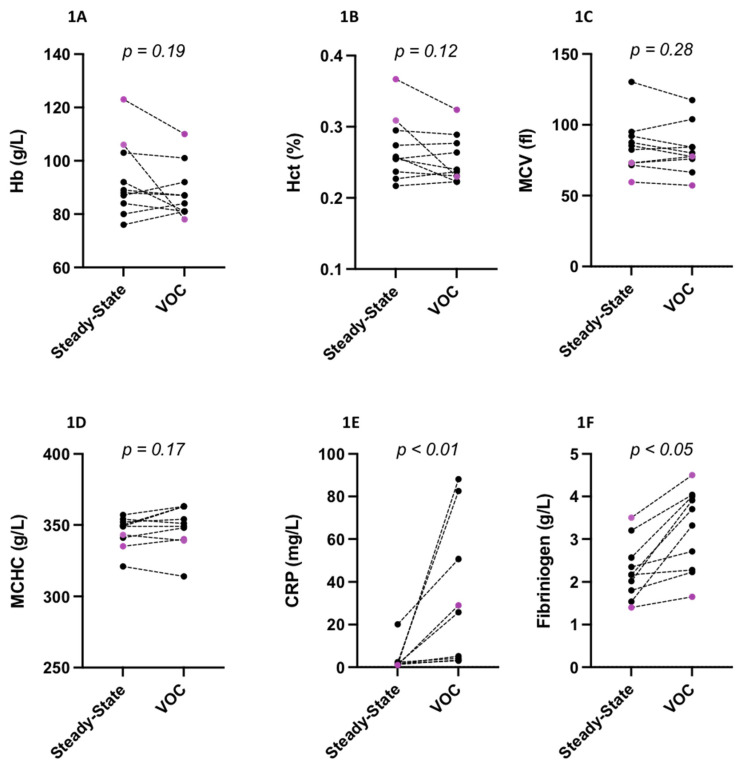
Hemoglobin (Hb, **1A**), hematocrit (Hct, **1B**), mean cell volume (MCV, **1C**), mean corpuscular hemoglobin concentration (MCHC, **1D**), C-reactive protein (CRP, **1E**) and fibrinogen (**1F**) levels in SCD patients at steady state and during vaso-occlusive crisis (VOC). Black = HbSS; purple = HbSC.

**Figure 2:**
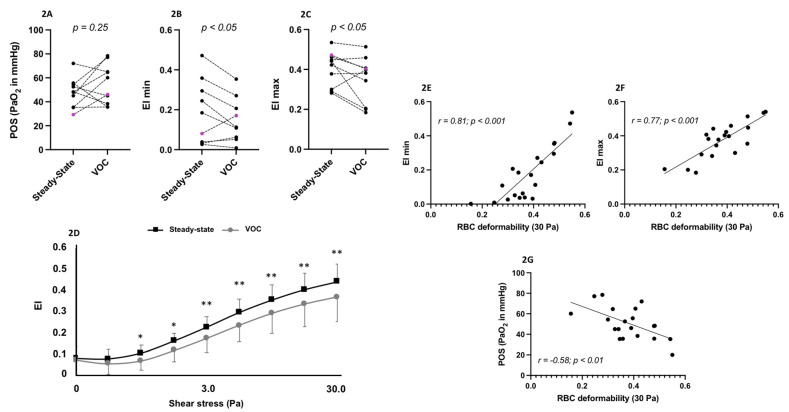
Point of sickling (PoS, **2A**), maximal RBC deformability in normoxia (EImax, **2B**), minimum RBC deformability during deoxygenation (EImin, **2C**) and shear-stress gradient RBC deformability (**2D**) in SCD patients at steady state and during vaso-occlusive crisis (VOC). Black = HbSS; purple = HbSC. Correlations between RBC deformability at 30 Pa and EImin (**2E**), EImax (**2F**) and PoS (**2G**) in SCD patients (steady state and VOC data are mixed). Difference between steady-state and VOC: * *p* < 0.05; ** *p* < 0.01.

Hb, Hct, MCV and MCHC were not different between steady state and VOC (Figure 1A–D) but CRP (Figure 1E) and fibrinogen (Figure 1F) levels increased during VOC. Figure 2A–C show the oxygenscan parameters: while EImin (2B) and EImax (2C) decreased, PoS remained unchanged between VOC and steady state (2A). Figure 2D shows the shear gradient ektacytometry RBC deformability values at the 9 increasing shear stresses: although no difference between VOC and steady state was observed at the two lowest shear stresses (0.3 and 0.53 Pa), RBC deformability was lower during VOC at the seven other shear stresses. Mixing steady state and VOC values show correlations between RBC deformability at 30 Pa and EImin (r = 0.81; *p* < 0.001; Figure 2E), EImax (r = 0.77; *p* < 0.001; Figure 2F) and, to a lesser extent, PoS (r = −0.58; *p* < 0.01; Figure 2G).

### 3.2. Biological and Ektacytometry Parameters Stability

**Figure 3:**
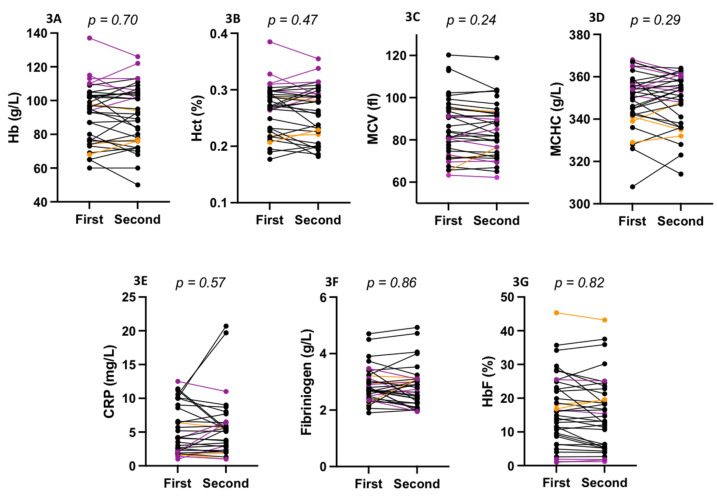
Hemoglobin (Hb, **3A**), hematocrit (Hct, **3B**), mean cell volume (MCV, **3C**), mean corpuscular hemoglobin concentration (MCHC, **3D**), C-reactive protein (CRP, **3E**), fibrinogen (**3F**) and fetal Hb (HbF; **3G**) levels measured in SCD patients at steady state on two different occasions. Black = HbSS; purple = HbSC; orange = HbSβ°.

**Figure 4:**
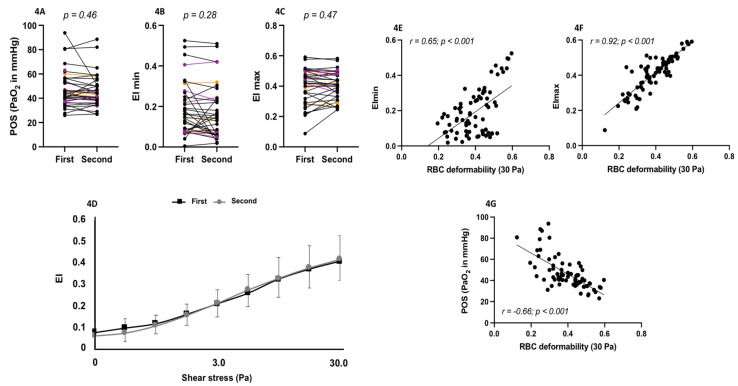
Point of sickling (PoS, **4A**), maximal RBC deformability in normoxia (EImax, **4B**), minimum RBC deformability during deoxygenation (EImin, **4C**) and shear-stress gradient RBC deformability (**4D**) measured in SCD patients at steady state on two different occasions. Black = HbSS; Purple = HbSC; Orange = HbSβ°. Correlations between RBC deformability at 30 Pa and EImin (**4E**), EImax (**4F**) and PoS (**4G**) in SCD patients (first and second measurements are mixed).

**Figure 5:**
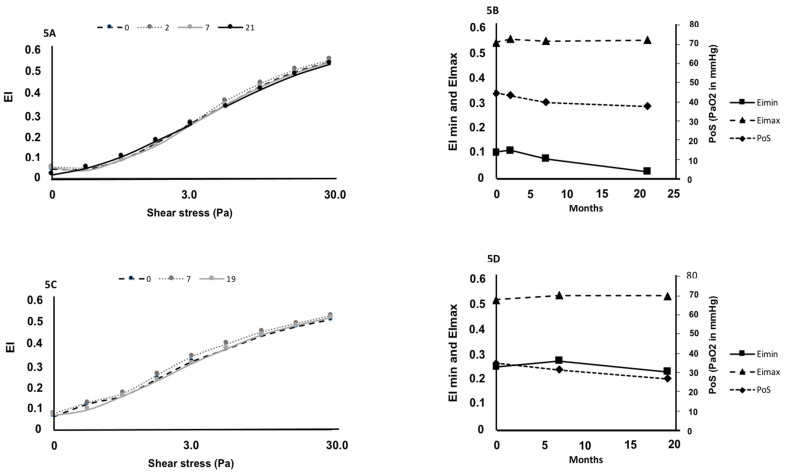
Shear-stress-gradient ektacytometry (**5A**,**5C**) and oxygenscan (**5B**,**5D**) parameters measured in two SCD patients at steady state on several occasions. For patient 1, measurements were performed on four different occasions: 0 = first measurement; 2, 7 and 21 = 2, 7 and 21 months after the first measurement, respectively. For patient 2, measurements were performed on three different occasions: 0 = first measurement; 7 and 19 = 7 and 19 months after the first measurement, respectively.

Hematological (Figure 3A–D), biochemical (Figure 3E–F), fetal Hb (HbF), oxygenscan (Figure 4A–C) and shear-stress-gradient ektacytometry (Figure 4D) parameters did not change between the first and the second measurement in SCD patients at steady state. Figure 5 shows the shear-stress-gradient ektacytometry (5A,5C) and oxygenscan (5B,5D) values for two patients having more than two repeated measurements over almost a 2-year period. The different parameters were rather stable over time. When mixing the data obtained at the two separate times, RBC deformability at 30 Pa correlated with EImin (r = 0.65; *p* < 0.001; Figure 4E), EImax (r = 0.92; *p* < 0.001; Figure 4F) and with PoS (r = −0.66; *p* < 0.001; Figure 4G).

## 4. Discussion

The clinical expression of SCD is highly variable from one patient to another and may change over time for a given patient. Indeed, the occurrence of acute and chronic complications are still unpredictable in most patients, and relevant and accurate biomarkers need to be developed and validated to improve clinical management [26]. SCD is a blood rheological disease characterized by a profound reduction in RBC deformability [2]. Various techniques have been used to measure RBC deformability or RBC membrane stiffness in SCD, including micropipette aspiration [27,28], filtration [29], atomic force microscopy [30], optical tweezers [31], microfluidic systems [32,33] and ektacytometry [1,34,35,36], among others. 

Osmolality ektacytometry is by far the most frequent method used to detect RBC membrane disorders such as hereditary spherocytosis, ovalocytosis, stomatocytosis and others [37,38]. The technique consists of measuring the changes in RBC deformability while osmolality varies to force the cells to swell (hypotonic suspension) and shrink (hypertonic suspension) [1]. The resulting changes in RBC deformability are depicted in a curve called osmoscan, which allows the determination of several key parameters reflecting the osmotic fragility, hydration status and cell surface/area ratio of the RBCs [1,37]. While this method is primarily used for the diagnosis of RBC-related membrane disorders, it has also been used in the context of SCD; however, its interpretation is rather complex [37]. Nevertheless, osmoscan studies have demonstrated a decreased RBC deformability in SCD patients compared to controls, with a further reduction during VOC [34,35] and higher RBC deformability in patients taking hydroxyurea compared to those who were not [39]. 

Shear-stress-gradient ektacytometry is the most frequent method used by scientists working in the field of blood rheology [22], and studies performed in the last 15 years in SCD have focused on the association between RBC deformability and clinical severity using this method [2]. Although the technique provides only a mean index of RBC deformability that does not fully reflect the rheological heterogeneity of the blood suspension and is unable to measure deformability in individual cells [40], it has the advantage of not being time consuming, and of being usable in biological routine. It has been reported that RBC deformability is decreased at low, intermediate and high shear stresses in SCD patients compared to healthy controls [23,41], with a further reduction during VOC [42], as observed in the present study. A reduction in RBC deformability measured above 3 Pa suggests a decreased RBC surface-area-to-volume ratio and/or a loss of membrane elasticity and/or an increase in internal viscosity, while a reduction in RBC deformability measured at shear stresses lower than 3 Pa reflects a loss of membrane elasticity only [43]. Shear-stress-gradient ektacytometry works have shown that patients with the lowest RBC deformability values have a high rate of hemolysis and develop complications, such as priapism, leg ulcers, glomerulopathy or cerebral vasculopathy [8,9,10,11].

Compared to osmoscan and shear-stress-gradient ektacytometry where the ambient pO_2_ corresponds to a normoxic environment, oxygenscan has the advantage to measure the changes in RBC over a pO_2_ gradient, which allows the assessment of the RBC sickling process [17,19]. The present study shows that, like RBC deformability measured by shear-stress-gradient ektacytometry, EImin and EImax determined by oxygenscan are impacted during/by VOC. The results were less clear for the PoS. PoS has been demonstrated to be sensitive to treatment, such as hydroxyurea therapy, voxelotor therapy or transfusion, [16,17,20] and to SCD genotypes [16]. In addition, Rab et al. [17] reported a greater PoS at steady state in children and adults with frequent VOCs compared to those with a less severe phenotype, meaning that the RBCs of patients with frequent VOC are susceptible to sickle at greater pO_2_ than the RBCs of patients with few VOCs. Our results did not demonstrate a significant difference for PoS between acute VOC and steady state, which seems intriguing. One could hypothesize that the most abnormal RBCs during VOC would be occluding vessels, and therefore, they could not be investigated via blood sampling. However, the individual responses were very heterogenous from one patient to another, and studies including a larger group of patients would be needed to compare the PoS between VOC and steady state to better understand why there is a large increase in some patients (5 in 10 in the present study) and a decrease in others (4 in 10 in the present study). 

The occurrence of acute and chronic complications is difficult to predict in SCD, and it is indeed necessary to develop biomarkers of disease activity and progression [26]. Indeed, one would expect that such biomarkers would remain unchanged in cases of no disease progression. The two repeated measurements, separated on average by one year and a half, performed in 38 SCD patients at steady state and with stable disease (i.e., no change in their treatment or clinical management, no change in the clinical severity) demonstrated that RBC rheological parameters were rather stable over time. Neither shear-stress gradient RBC deformability nor oxygenscan parameters changed between the first and second measurements, as was also the case for the hematological and biochemical parameters. In contrast, the occurrence of VOC was accompanied by a change in RBC deformability, both in normoxic and hypoxic conditions, without any change in hematological parameters, showing that shear-stress-gradient ektacytometry and oxygenscan methods are able to provide biomarkers of disease activity. Nevertheless, like for the hematological and biochemical parameters, we noted some inter-individual differences in the RBC rheological behaviors between VOC and steady state, as well as between the first and second measurements at steady state; these justify the performance of larger studies such as this one to better understand the biological meaning of these differences, and to test the correlations with the clinical evolution of the patients. The measurements of CRP and fibrinogen levels demonstrated a stability of these parameters over time in stable patients and significant changes during acute complications. It is difficult to know if these biochemical parameters are better biomarkers of disease activity and clinical severity than RBC deformability to follow and predict the clinical evolution of SCD patients. CRP is the most widely used marker of acute and chronic inflammation in SCD, with high levels at steady state correlating with increased VOC frequency in children [44]. It has also been reported that CRP level might be useful to anticipate the development of acute chest syndrome [45]. However, CRP is not specific to SCD and is elevated in several acute and chronic inflammatory states [46]. In contrast, the measurement of RBC deformability at various shear stresses or over an oxygen gradient is more specific to SCD pathophysiology, and offers the advantage of providing markers that may be sensitive to current and candidate SCD therapies [16,17,20]. The recent development of specialized equipment may facilitate access to fast routine measurements of these RBC rheological parameters. However, although some efforts have been made to standardize the oxygenscan [24,25] and shear-stress-gradient ektacytometry methods [22,23], reproducibility of measurements between different laboratories needs to be assessed. The delay between blood sampling and measurements must also be standardized and reduced as much as possible, to avoid any changes in RBC rheology that could be related to blood storage and that would not reflect the disease activity [18,24,25,26]. Finally, international collaborations and prospective studies should be stimulated to help in determining the clinical usefulness of shear-stress-gradient ektacytometry and oxygenscan in large cohorts of SCD patients, and to demonstrate the superiority of such parameters over classical biomarkers of the disease such as hemoglobin concentration, HbF level and others; such collaborations and studies could also demonstrate the complementary nature of the incremental value of such parameters with the other classical parameters to better characterize the disease.

## 5. Conclusion

In conclusion, the present study showed that both oxygen gradient ektacytometry and shear-stress-gradient ektacytometry are sensitive to disease activity in SCD, and that both techniques give comparable results. The determination of the PoS during VOC gave intriguing results, with some patients exhibiting a decrease while others had an increase, in comparison with the values determined at steady state. Although all measurements were conducted in optimal conditions (i.e., on fresh samples after the onset of VOC), the sample size of the group was limited, and further longitudinal studies are needed to test the effects of VOC on PoS, as well as its clinical relevance. 

## Data Availability

Data are available upon reasonable request to the corresponding author.

## References

[1] Clark M.R., Mohandas N., Shohet S.B. (1980). Deformability of oxygenated irreversibly sickled cells. J. Clin. Invest..

[2] Connes P., Alexy T., Detterich J., Romana M., Hardy-Dessources M.D., Ballas S.K. (2016). The role of blood rheology in sickle cell disease. Blood Rev..

[3] Murayama M. (1966). Molecular mechanism of red cell “sickling”. Science.

[4] Lessin L.S., Kurantsin-Mills J., Wallas C., Weems H. (1978). Membrane alterations in irreversibly sickled cells: Hemoglobin--membrane interaction. J. Supramol. Struct..

[5] Lux S.E., John K.M., Karnovsky M.J. (1976). Irreversible deformation of the spectrin-actin lattice in irreversibly sickled cells. J. Clin. Invest..

[6] Brugnara C., Tosteson D.C. (1987). Inhibition of K transport by divalent cations in sickle erythrocytes. Blood.

[7] Connes P., Lamarre Y., Waltz X., Ballas S.K., Lemonne N., Etienne-Julan M., Hue O., Hardy-Dessources M.D., Romana M. (2014). Haemolysis and abnormal haemorheology in sickle cell anaemia. Br. J. Haematol..

[8] Brousse V., Pondarre C., Kossorotoff M., Arnaud C., Kamdem A., de Montalembert M., Boutonnat-Faucher B., Allali S., Bourdeau H., Charlot K. (2021). Brain injury pathophysiology study by a multimodal approach in children with sickle cell anemia with no intra or extra cranial arteriopathy. Haematologica.

[9] Cita K.C., Brureau L., Lemonne N., Billaud M., Connes P., Ferdinand S., Tressieres B., Tarer V., Etienne-Julan M., Blanchet P. (2016). Men with Sickle Cell Anemia and Priapism Exhibit Increased Hemolytic Rate, Decreased Red Blood Cell Deformability and Increased Red Blood Cell Aggregate Strength. PLoS ONE.

[10] Connes P., Lamarre Y., Hardy-Dessources M.D., Lemonne N., Waltz X., Mougenel D., Mukisi-Mukaza M., Lalanne-Mistrih M.L., Tarer V., Tressieres B. (2013). Decreased hematocrit-to-viscosity ratio and increased lactate dehydrogenase level in patients with sickle cell anemia and recurrent leg ulcers. PLoS ONE.

[11] Lamarre Y., Romana M., Lemonne N., Hardy-Dessources M.D., Tarer V., Mougenel D., Waltz X., Tressieres B., Lalanne-Mistrih M.L., Etienne-Julan M. (2014). Alpha thalassemia protects sickle cell anemia patients from macro-albuminuria through its effects on red blood cell rheological properties. Clin. Hemorheol. Microcirc..

[12] Lamarre Y., Romana M., Waltz X., Lalanne-Mistrih M.L., Tressieres B., Divialle-Doumdo L., Hardy-Dessources M.D., Vent-Schmidt J., Petras M., Broquere C. (2012). Hemorheological risk factors of acute chest syndrome and painful vaso-occlusive crisis in children with sickle cell disease. Haematologica.

[13] Renoux C., Connes P., Nader E., Skinner S., Faes C., Petras M., Bertrand Y., Garnier N., Cuzzubbo D., Divialle-Doumdo L. (2017). Alpha-thalassaemia promotes frequent vaso-occlusive crises in children with sickle cell anaemia through haemorheological changes. Pediatr Blood Cancer.

[14] Charlot K., Romana M., Moeckesch B., Jumet S., Waltz X., Divialle-Doumdo L., Hardy-Dessources M.D., Petras M., Tressieres B., Tarer V. (2016). Which side of the balance determines the frequency of vaso-occlusive crises in children with sickle cell anemia: Blood viscosity or microvascular dysfunction?. Blood Cells Mol. Dis..

[15] Nebor D., Bowers A., Hardy-Dessources M.D., Knight-Madden J., Romana M., Reid H., Barthelemy J.C., Cumming V., Hue O., Elion J. (2011). Frequency of pain crises in sickle cell anemia and its relationship with the sympatho-vagal balance, blood viscosity and inflammation. Haematologica.

[16] Boisson C., Rab M.A.E., Nader E., Renoux C., Kanne C., Bos J., van Oirschot B.A., Joly P., Fort R., Gauthier A. (2021). Effects of Genotypes and Treatment on Oxygenscan Parameters in Sickle Cell Disease. Cells.

[17] Rab M.A.E., Kanne C.K., Bos J., van Oirschot B.A., Boisson C., Houwing M.E., Gerritsma J., Teske E., Renoux C., Riedl J. (2021). Oxygen gradient ektacytometry-derived biomarkers are associated with vaso-occlusive crises and correlate with treatment response in sickle cell disease. Am. J. Hematol..

[18] Sadaf A., Seu K.G., Thaman E., Fessler R., Konstantinidis D.G., Bonar H.A., Korpik J., Ware R.E., McGann P.T., Quinn C.T. (2021). Automated Oxygen Gradient Ektacytometry: A Novel Biomarker in Sickle Cell Anemia. Front. Physiol..

[19] Rab M.A.E., van Oirschot B.A., Bos J., Merkx T.H., van Wesel A.C.W., Abdulmalik O., Safo M.K., Versluijs B.A., Houwing M.E., Cnossen M.H. (2019). Rapid and reproducible characterization of sickling during automated deoxygenation in sickle cell disease patients. Am. J. Hematol..

[20] Chonat S., Fields E., Baratz H., Watt A., Pochron M., Dixon S., Tonda M., Lehrer-Graiwer J., Brown C., Archer D.R. (2019). Improvement in Red Blood Cell Physiology in Children with Sickle Cell Anemia Receiving Voxelotor. Blood.

[21] Hierso R., Lemonne N., Villaescusa R., Lalanne-Mistrih M.L., Charlot K., Etienne-Julan M., Tressieres B., Lamarre Y., Tarer V., Garnier Y. (2017). Exacerbation of oxidative stress during sickle vaso-occlusive crisis is associated with decreased anti-band 3 autoantibodies rate and increased red blood cell-derived microparticle level: A prospective study. Br. J. Haematol..

[22] Baskurt O.K., Boynard M., Cokelet G.C., Connes P., Cooke B.M., Forconi S., Liao F., Hardeman M.R., Jung F., Meiselman H.J. (2009). New guidelines for hemorheological laboratory techniques. Clin. Hemorheol. Microcirc..

[23] Renoux C., Parrow N., Faes C., Joly P., Hardeman M., Tisdale J., Levine M., Garnier N., Bertrand Y., Kebaili K. (2016). Importance of methodological standardization for the ektacytometric measures of red blood cell deformability in sickle cell anemia. Clin. Hemorheol. Microcirc..

[24] Boisson C., Rab M.A.E., Nader E., Renoux C., van Oirschot B.A., Joly P., Fort R., Stauffer E., van Beers E.J., Sheehan V.A. (2021). Methodological aspects of oxygen gradient ektacytometry in sickle cell disease: Effects of sample storage on outcome parameters in distinct patient subgroups. Clin. Hemorheol. Microcirc..

[25] Rab M.A.E., Kanne C.K., Bos J., Boisson C., van Oirschot B.A., Nader E., Renoux C., Joly P., Fort R., van Beers E.J. (2020). Methodological aspects of the oxygenscan in sickle cell disease: A need for standardization. Am. J. Hematol..

[26] Rees D.C., Gibson J.S. (2012). Biomarkers in sickle cell disease. Br. J. Haematol..

[27] Havell T.C., Hillman D., Lessin L.S. (1978). Deformability characteristics of sickle cells by microelastimetry. Am. J. Hematol..

[28] Nash G.B., Johnson C.S., Meiselman H.J. (1986). Influence of oxygen tension on the viscoelastic behavior of red blood cells in sickle cell disease. Blood.

[29] Reid H.L., Obi G.O. (1982). A study of erythrocyte deformability in sickle cell disease. Trop. Geogr. Med..

[30] Maciaszek J.L., Lykotrafitis G. (2011). Sickle cell trait human erythrocytes are significantly stiffer than normal. J. Biomech..

[31] Brandao M.M., Fontes A., Barjas-Castro M.L., Barbosa L.C., Costa F.F., Cesar C.L., Saad S.T. (2003). Optical tweezers for measuring red blood cell elasticity: Application to the study of drug response in sickle cell disease. Eur J. Haematol..

[32] Alapan Y., Matsuyama Y., Little J.A., Gurkan U.A. (2016). Dynamic deformability of sickle red blood cells in microphysiological flow. Technol. (Singap. World Sci.).

[33] Faivre M., Renoux C., Bessaa A., Da Costa L., Joly P., Gauthier A., Connes P. (2020). Mechanical Signature of Red Blood Cells Flowing Out of a Microfluidic Constriction Is Impacted by Membrane Elasticity, Cell Surface-to-Volume Ratio and Diseases. Front. Physiol..

[34] Ballas S.K. (1991). Sickle cell anemia with few painful crises is characterized by decreased red cell deformability and increased number of dense cells. Am. J. Hematol..

[35] Ballas S.K., Larner J., Smith E.D., Surrey S., Schwartz E., Rappaport E.F. (1988). Rheologic predictors of the severity of the painful sickle cell crisis. Blood.

[36] Embury S.H., Clark M.R., Monroy G., Mohandas N. (1984). Concurrent sickle cell anemia and alpha-thalassemia. Effect on pathological properties of sickle erythrocytes. J. Clin. Invest..

[37] Clark M.R., Mohandas N., Shohet S.B. (1983). Osmotic gradient ektacytometry: Comprehensive characterization of red cell volume and surface maintenance. Blood.

[38] Da Costa L., Galimand J., Fenneteau O., Mohandas N. (2013). Hereditary spherocytosis, elliptocytosis, and other red cell membrane disorders. Blood Rev..

[39] Ballas S.K., Connes P., Investigators of the Multicenter Study of Hydroxyurea in Sickle Cell Anemia (2018). Rheological properties of sickle erythrocytes in patients with sickle-cell anemia: The effect of hydroxyurea, fetal hemoglobin, and alpha-thalassemia. Eur. J. Haematol..

[40] Parrow N.L., Violet P.C., Tu H., Nichols J., Pittman C.A., Fitzhugh C., Fleming R.E., Mohandas N., Tisdale J.F., Levine M. (2018). Measuring Deformability and Red Cell Heterogeneity in Blood by Ektacytometry. J. Vis. Exp..

[41] Tripette J., Alexy T., Hardy-Dessources M.D., Mougenel D., Beltan E., Chalabi T., Chout R., Etienne-Julan M., Hue O., Meiselman H.J. (2009). Red blood cell aggregation, aggregate strength and oxygen transport potential of blood are abnormal in both homozygous sickle cell anemia and sickle-hemoglobin C disease. Haematologica.

[42] Lapoumeroulie C., Connes P., El Hoss S., Hierso R., Charlot K., Lemonne N., Elion J., Le Van Kim C., Romana M., Hardy-Dessources M.D. (2019). New insights into red cell rheology and adhesion in patients with sickle cell anaemia during vaso-occlusive crises. Br. J. Haematol..

[43] Renoux C., Faivre M., Bessaa A., Da Costa L., Joly P., Gauthier A., Connes P. (2019). Impact of surface-area-to-volume ratio, internal viscosity and membrane viscoelasticity on red blood cell deformability measured in isotonic condition. Sci. Rep..

[44] Krishnan S., Setty Y., Betal S.G., Vijender V., Rao K., Dampier C., Stuart M. (2010). Increased levels of the inflammatory biomarker C-reactive protein at baseline are associated with childhood sickle cell vasocclusive crises. Br. J. Haematol..

[45] Bargoma E.M., Mitsuyoshi J.K., Larkin S.K., Styles L.A., Kuypers F.A., Test S.T. (2005). Serum C-reactive protein parallels secretory phospholipase A2 in sickle cell disease patients with vasoocclusive crisis or acute chest syndrome. Blood.

[46] Pepys M.B., Hirschfield G.M. (2003). C-reactive protein: A critical update. J. Clin. Invest..

